# Clinical characteristics of tumor-induced osteomalacia lesions in the neurocranium and intracranial structures

**DOI:** 10.1186/s13023-026-04477-1

**Published:** 2026-07-07

**Authors:** Yushuo Wu, Xiang Li, Zhiqin Xu, Wei Lv, Zhixiang Wang, Xiaolin Ni, Qianqian Pang, Ruizhi Jiajue, Yue Chi, Wei Liu, Lijia Cui, Yan Jiang, Ou Wang, Mei Li, Xiaoping Xing, Wenbin Ma, Yu Wang, Yong Yao, Yi Guo, Zhiqiang Gao, Guodong Feng, Fang Qi, Yiyi Gong, Weibo Xia

**Affiliations:** 1 Department of Endocrinology, NHC Key Laboratory of Endocrinology, Peking Union Medical College Hospital, Chinese Academy of Medical Sciences & Peking Union Medical College, No. 1 Shuaifuyuan, Dongcheng District, Beijing, 100730 China; 2 State Key Laboratory of Complex, Severe, and Rare Diseases, Institute of Clinical Medicine, Peking Union Medical College Hospital, Chinese Academy of Medical Sciences & Peking Union Medical College, Beijing, 100730 China; 3 Department of Neurosurgery, Peking Union Medical College Hospital, Chinese Academy of Medical Sciences and Peking Union Medical College, Beijing, 100730 China; 4 Department of Otorhinolaryngology, Peking Union Medical College Hospital, Chinese Academy of Medical Sciences and Peking Union Medical College, Beijing, 100730 China; 5 Department of Endocrinology, Genetics and Metabolism, Beijing Children’s Hospital, Capital Medical University, National Center for Children’s Health, Beijing, 100045 China; 6 Center for Biomarker Discovery and Validation, Institute of Clinical Medicine, National Infrastructures for Translational Medicine (PUMCH), Peking Union Medical College Hospital, Chinese Academy of Medical Sciences and Peking Union Medical College, No. 1 Shuaifuyuan, Dongcheng District, Beijing, 100730 China

**Keywords:** Tumor-induced osteomalacia, Lesions in the neurocranium and intracranial structures, Clinical characteristics

## Abstract

**Background:**

Tumor-induced osteomalacia (TIO) is a rare paraneoplastic syndrome caused by excessive fibroblast growth factor 23 (FGF23) secretion. However, TIO lesions located in the neurocranium (NC) and intracranial (IC) structures remain poorly characterized.

**Objective:**

To characterize the clinical features of TIO lesions in NC and IC structures and to compare them with those located in the sinuses.

**Methods:**

This retrospective study included patients diagnosed with TIO who underwent tumor resection at Peking Union Medical College Hospital between January 2016 and December 2023. Patients with lesions in NC and IC structures were defined as the study group, while those with sinus lesions served as the control group. Clinical characteristics, biochemical parameters, and surgical outcomes were systematically analyzed and compared between groups.

**Results:**

TIO lesions in NC and IC structures were distributed across the skull base, temporal region, sellar region, frontal lobe, and occipital region. Compared with sinus lesions, patients with NC and IC lesions showed comparable clinical and biochemical profiles but exhibited significantly worse outcomes, including higher rates of non-remission and tumor recurrence. These findings suggest increased difficulty in complete tumor resection and disease control in this subgroup.

**Conclusion:**

TIO lesions in NC and IC structures are associated with poorer surgical outcomes and higher recurrence rates despite similar baseline characteristics. These results highlight the need for improved localization strategies and tailored management approaches for this challenging subgroup.

**Supplementary Information:**

The online version contains supplementary material available at https://doi.org/10.1186/s13023-026-04477-1.

## Introduction

Tumor-induced osteomalacia (TIO) is the most prevalent form of acquired hypophosphatemic osteomalacia but a rare paraneoplastic syndrome [1, 2]. Tumors classified as phosphaturic mesenchymal tumors (PMTs) produce excessive amounts of fibroblast growth factor 23 (FGF23), leading to reduced renal phosphate reabsorption, hypophosphatemia, and suppression of 1,25-dihydroxyvitamin D [1,25(OH)2D] production. [2, 3]. TIO patients often suffer from progressive and severe muscle weakness, bone pain, impaired mobility, and repeating fragility fractures.

Tumor-induced osteomalacia (TIO) is a disease that can be cured through surgical intervention. However, the cure rate is closely related to whether the lesions’ location allow for complete surgical removal. A previous study found that the refractory rate of TIO lesions in the head and neck, including those in the neurocranium (NC) and intracranial (IC) structures, in the sinuses, and in the upper and lower jaws, were lower compared to lesions in other sites [4]. TIO lesions located above the skull, due to their unique position, may differ from the other head and neck TIO lesions. They might present distinct clinical features and potentially pose greater treatment challenges. However, there is currently no study that summarizes the characteristics of this type of TIO lesions. Therefore, the purpose of this study was to summarize the characteristics of TIO lesions in the NC and IC structures in the largest Chinese TIO cohort to date to deepen the understanding of this type of TIO lesions.

## Patients and methods

### Study population

All lesions originating from the anatomical area in the NC and IC structures, including the skull base itself, were considered as lesions in the NC and IC structures. Patients with lesions in the paranasal sinuses including the ethmoid, sphenoid, frontal, and maxillary sinuses were classified as the control group because their anatomical location was adjacent to lesions in the NC and IC structures. Lesions in the sinuses that extended to the ethmoid bone, the sphenoid bone, and the skull base were still classified as sinus lesions.

Patients with TIO lesions in the NC and IC structures and lesions in the sinuses who visited and underwent tumor excision surgery at Peking Medical College Hospital (PUMCH) between January 1, 2016 and December 31, 2023 were enrolled in this study.

These TIO patients had three outcomes: nonremission, remission, and relapse. Postoperative serum phosphorus levels of nonremission patients did not return to normal levels. In contrast, postoperative serum phosphorus levels of remission patients normalized and remained normal during follow-up (follow-up time ≥ 3 months). For patients with relapse outcomes, serum phosphorus levels initially returned to normal but hypophosphatemia recurred during follow-up (follow-up time ≥ 3 months). Nonremission and relapse outcomes were considered refractory.

This study was approved by the Local Ethics Committee of the Department of Scientific Research at PUMCH, and informed consent was obtained from all participants before enrollment in the study. All procedures were conducted in accordance with the Declaration of Helsinki.

### General clinical characteristics and histopathology

Sex, age of onset, disease duration, height loss, skeleton deformity, abnormal gait, history of fracture, preoperative compression symptoms, postoperative complications were collected from the patients’clinical records.

Histologic results were also extracted from the clinical records. Immunohistochemical staining was conducted for differential diagnosis within the Pathology Department. The results were interpreted by senior pathologists to established the definitive pathological diagnosis.

### Biochemical measurements

Serum calcium, phosphate, and alkaline phosphatase (ALP) levels were measured by an autoanalyzer (AU5800, Beckman Coulter, Brea, CA, USA). Serum intact parathyroid hormone (iPTH) levels were measured by an autoanalyzer (DXI800, Beckman Coulter, America; Beckman Coulter Cat# A16972, RRID: *AB_2924840*). 25-hydroxy vitamin D (25OHD), beta-C-terminal telopeptide of type I collagen (β-CTX) levels were measured by an automated Roche electrochemiluminescence system (E601, Roche Diagnostics, Basel, Switzerland). Serum 1,25(OH)_2_D levels were measured by radioimmunoassay/enzyme-linked immunoassay (DiaSorin, Stillwater, MN, USA). Urine was estimated by a urinary chemical analyzer (Clinitek 500, Siemens, Chicago, IL, USA). Renal tubular reabsorption of phosphate (TRP) was calculated using the following formula: %TRP = 100*[1–(urine phosphate*serum creatinine)/(serum phosphate*urine creatinine)]. TmP/GFR (tubular maximum reabsorption threshold of phosphate per glomerular filtration rate) was estimated using the Walton and Bijvoet nomogram [5]. Serum intact FGF23 (iFGF23) was measured by an iFGF23 ELISA Kit (Kainos Laboratories Cat# CY-4000, RRID: *AB_3105844*) as previously described [6].

### Tumor localization

All of patients suspected of having TIO underwent ^99m^Tc-OCT, which were executed by the Department of Nuclear Medicine of Peking Union Medical College Hospital (PUMCH). Upon the identification of suspicious lesions, anatomical imaging which included ultrasonography, computed tomography (CT), and magnetic resonance imaging (MRI) was conducted at the specified locations. The ultrasonography was carried out by the Ultrasound Department of PUMCH, while the CT and MRI scans were executed by the Radiology Department of PUMCH.The negative results of anatomical imaging of the specified locations were defined as the false positive results of ^99m^Tc-OCT. Patients with the negative or false positive results ^99m^Tc-OCT and some of patients with the positive results of ^99m^Tc-OCT underwent ^68^Ga-DOTATATE-PET/CT, which were also executed by the Department of Nuclear Medicine of Peking Union Medical College Hospital (PUMCH).

### Statistical analyses

Continuous variables were depicted as mean ± standard deviation (SD) or median (interquartile range, IQR) and categorical variables were expressed in terms of frequency (proportion). Comparative analysis between two groups was conducted using the independent samples *t* test for continuous data exhibiting normal distribution, the Mann-Whitney *U* test for non-normal continuous data, and the chi-square test for categorical data. *P* < 0.05 was considered statistically significant for two-tailed tests. All statistical analyses were conducted by IBM SPSS Statistical Software, version 25 (SPSS Inc., Chicago, IL, USA).

## Results

### General clinical characteristics and biochemical parameters

All general clinical characteristics and biochemical parameters of TIO patients with lesions in the NC and IC structures and lesions in the sinuses were listed in Table 1. There was no significant difference between general clinical characteristics of TIO patients with lesions in the NC and IC structures and lesions in the sinuses. No significant difference of biochemical parameters was found between TIO patients with lesions in the NC and IC structures and lesions in the sinuses.

**Table 1:** General clinical characteristics and biochemical parameters

	Lesions in the NC and IC structures	Lesions in the sinuses	*P*
Sex (male), *n* (%)	9 (69.2%)	24 (55.8%)	0.389
Age of onset (y)	36.33 (31.00, 46.75)	38.50 (33.42, 47.00)	0.749
Disease duration (y)	2.00 (1.16, 4.75)	3.25 (1.67, 6.00)	0.490
Height loss, n (%)	5 (50%)	27 (67.5%)	0.302
Heigh loss (cm)	1.0 (0.0, 3.5)	4.0 (0.0, 10.0)	0.102
Skeleton deformity, n (%)	5 (45.5%)	18 (42.9%)	0.877
Abnormal gait, n (%)	13 (100%)	41 (97.6%)	0.574
History of fracture, n (%)	8 (66.7%)	33 (78.6%)	0.395
Cr (µmol/L)	61.17 ± 13.76	59.14 ± 13.45	0.650
Pi (mmol/L)	0.48 ± 0.11	0.47 ± 0.11	0.685
Ca (mmol/L)	2.30 ± 0.10	2.28 ± 0.10	0.629
ALP (U/L)	290.0 (165.0, 374.0)	269.0 (194.0, 394.0)	0.621
iPTH (pg/ml)	48.4 (37.6, 94.6)	59.1 (41.9, 74.1)	0.459
25(OH)D (ng/ml)	17.95 ± 5.54	17.57 ± 7.03	0.863
1,25(OH)_2_D (pg/ml)	11.88 (0.01, 30.98)	7.47 (4.70, 19.73)	0.703
β-CTX (ng/ml)	0.60 (0.48, 0.83)	0.72 (0.40, 1.24)	0.710
24hUP (mmol/24 h)	15.47 (14.24, 23.28)	16.50 (12.45, 19.20)	0.615
24hUCa (mmol/24 h)	3.67 (2.27, 4.21)	2.36 (1.55, 4.10)	0.190
TRP (%)	85.2 (78.5, 90.8)	82.5 (77.0, 87.5)	0.327
TmP/GFR (mmol/L)	0.49 (0.34, 0.57)	0.45 (0.32, 0.53)	0.536
iFGF23 (pg/mL)	253.08 (188.94, 540.08)	205.45 (105.37, 323.75)	0.107

Normally distributed continuous variables were depicted as mean ± standard deviation (SD) and non-normally distributed continuous variables were shown as median (interquartile range, IQR). Categorical variables were expressed as counts (proportion). *P* values were obtained by the independent samples *t* test for normally distributed data or the Mann-Whitney *U* test for nonparametric data. Bold values denoted statistically significant differences (*P* < 0.05)

Abbreviations: Cr, serum creatinine; Pi, serum phosphate; Ca, serum total calcium; ALP, serum alkaline phosphatase; iPTH, serum intact parathyroid hormone; 25(OH)D, 25-hydroxy vitamin D; 1,25(OH)2D,1,25-dihydroxyvitamin D; β-CTX, beta-C-terminal cross-linking telopeptide of type I collagen; 24hUP, urine phosphate for 24 h; 24hUCa, urine calcium for 24 h; TRP,  renal tubular reabsorption of phosphate; TmP/GFR,  renal tubular maximum transport of phosphate (TMP) to glomerular filtration rate (GFR) ratio; iFGF23,  serum intact fibroblast growth factor 23

### Preoperative compression symptoms and postoperative complications

**Fig. 1:**
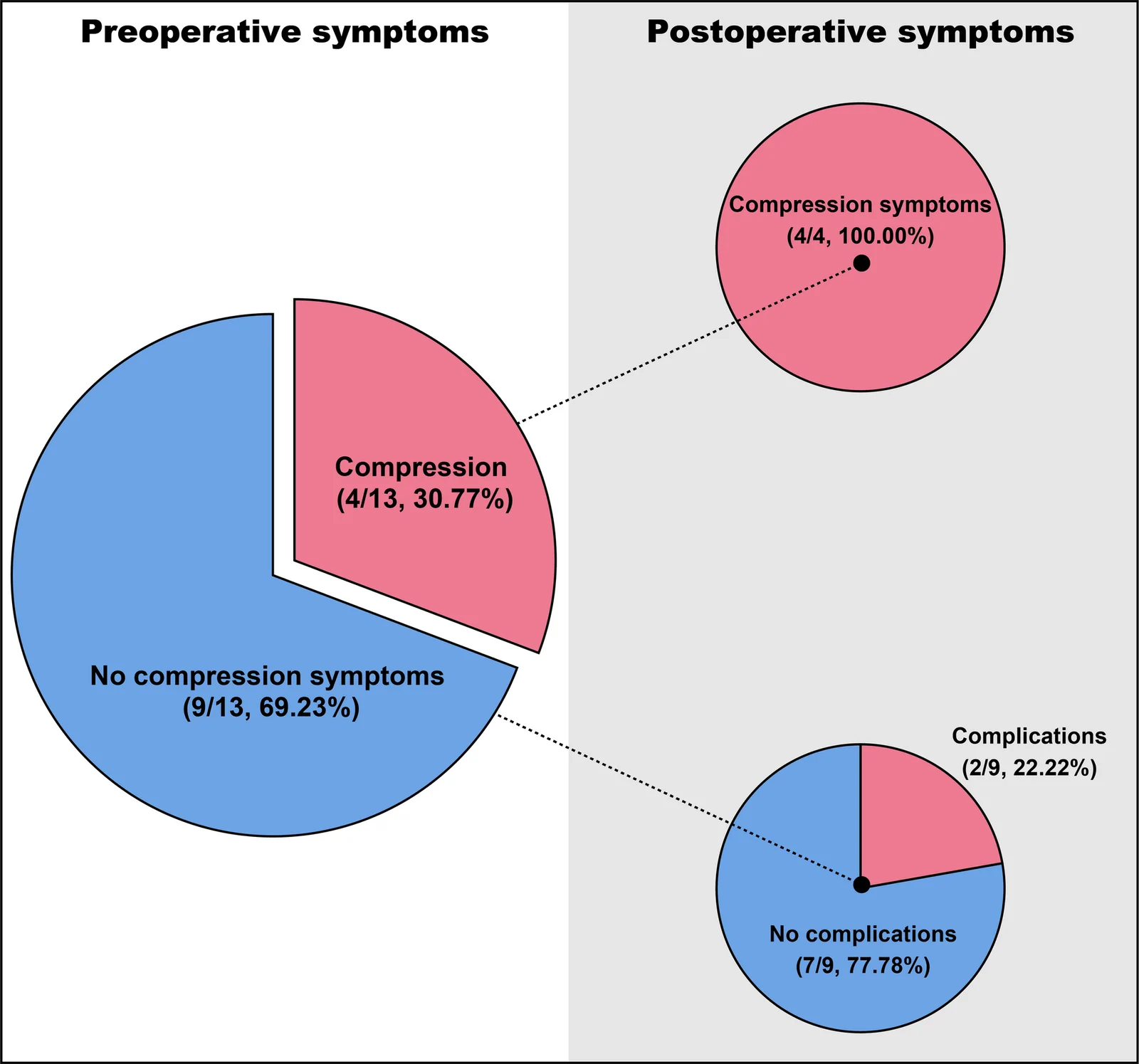
Preoperative compression symptoms and postoperative complications of TIO patients with lesions in the NC and IC structures

30.8% TIO patients with lesions in the NC and IC structures had compression symptoms such as hearing loss, limited eye movement, anterior pituitary hypofunction before being diagnosed as TIO. None of their compression symptoms recovered after operations. After operations, another two TIO patients with lesions in the NC and IC structures had postoperative complications such as hearing loss and facial paralysis because of the specific locations of the tumors (Fig. 1).

### Functional localization with 99mTc-OCT and 68Ga-DOTA-TATE PET/CT

**Fig. 2:**
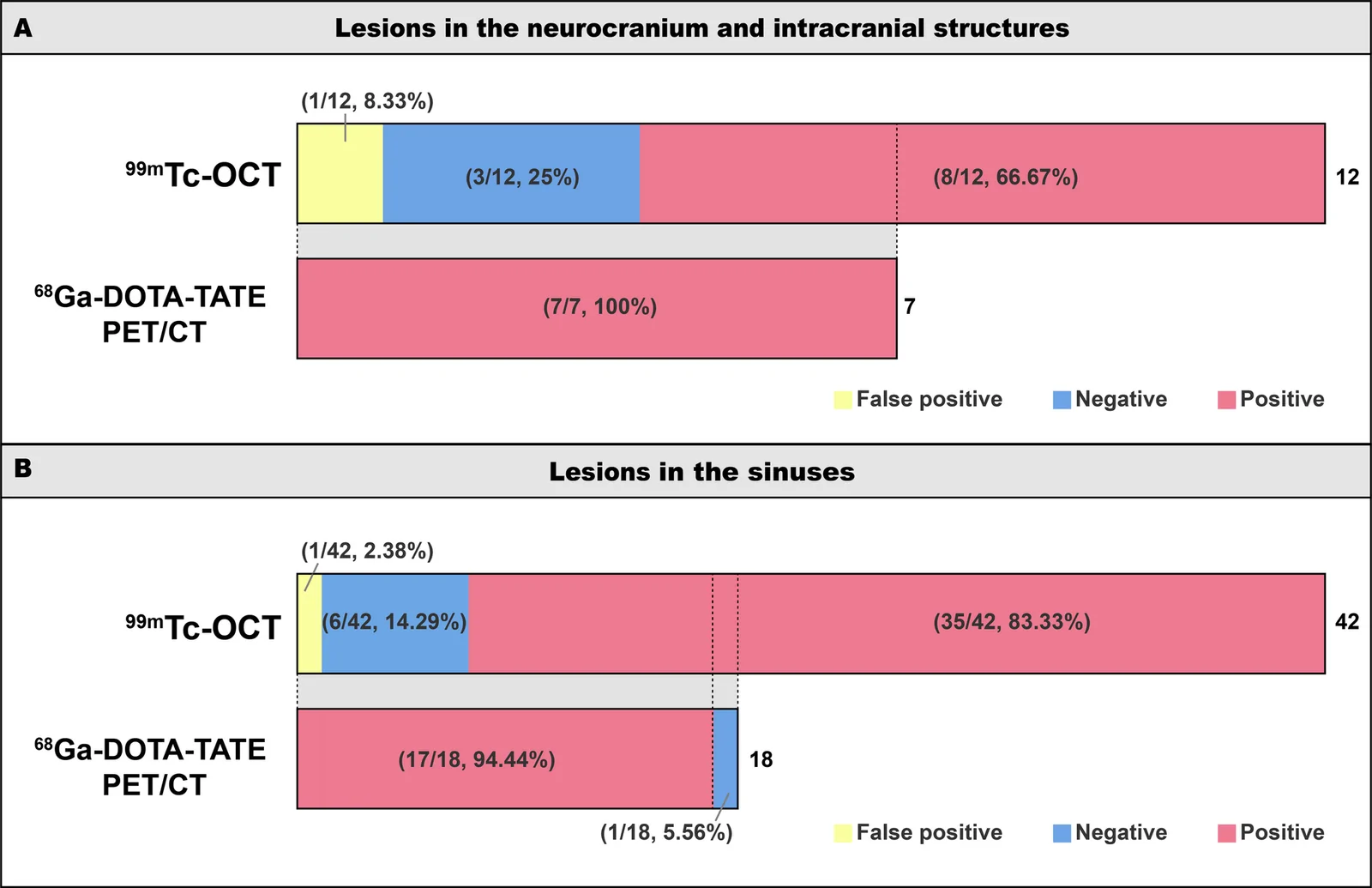
Outcomes of ^99m^Tc-OCT and ^68^Ga-DOTA-TATE PET/CT. (**A**) Outcomes of ^99m^Tc-OCT and ^68^Ga-DOTA-TATE PET/CT of TIO patients with lesions in the NC and IC structures. (**B**) Outcomes of ^99m^Tc-OCT and ^68^Ga-DOTA-TATE PET/CT of TIO patients with lesions in the sinuses. Abbreviations: ^99m^Tc-OCT, Technetium 99m octreotide scintigraphy; PET, Positron emission tomography; CT, Computed tomography

**Fig. 3:**
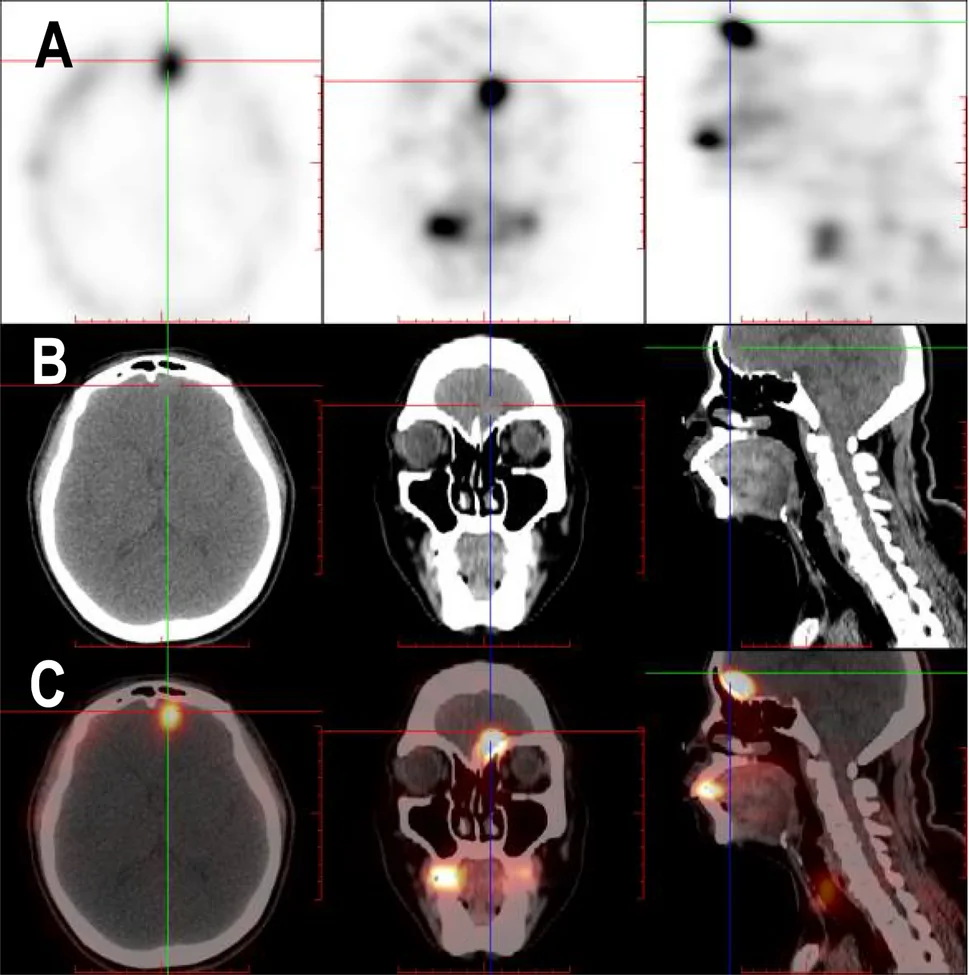
The ^99m^Tc-OCT outcome of one of TIO patients with lesions in the NC and IC structures. (**A**) SPECT imaging showing increased tracer uptake (**B**) CT imaging showing anatomical structures of NC and IC (**C**) Fused SPECT/CT imaging showing co-localization of uptake and anatomy

TIO patients with lesions in the sinuses (83.3%) had higher positive rate of the ^99m^Tc-OCT than TIO patients with lesions in the NC and IC structures (66.7%). Seven TIO patients with lesions in the NC and IC structures underwent ^68^Ga-DOTA-TATE PET/CT, including four with negative or false positive ^99m^Tc-OCT results and three with positive ^99m^Tc-OCT results. Eighteen patients with lesions in the sinuses underwent ^68^Ga-DOTA-TATE PET/CT, including seven with negative or false positive ^99m^Tc-OCT results and eleven with positive ^99m^Tc-OCT results. The positive rate of ^68^Ga-DOTA-TATE PET/CT in TIO patients with lesions in the NC and IC structures (100%) was a little higher than TIO patients with lesions in the sinuses (94.4%) (Fig. 2). The image of ^99m^Tc-OCT of one of TIO patients with lesions in the NC and IC structures was shown in Fig. 3.

### Distribution of TIO tumor in the NC and IC structures

**Fig. 4:**
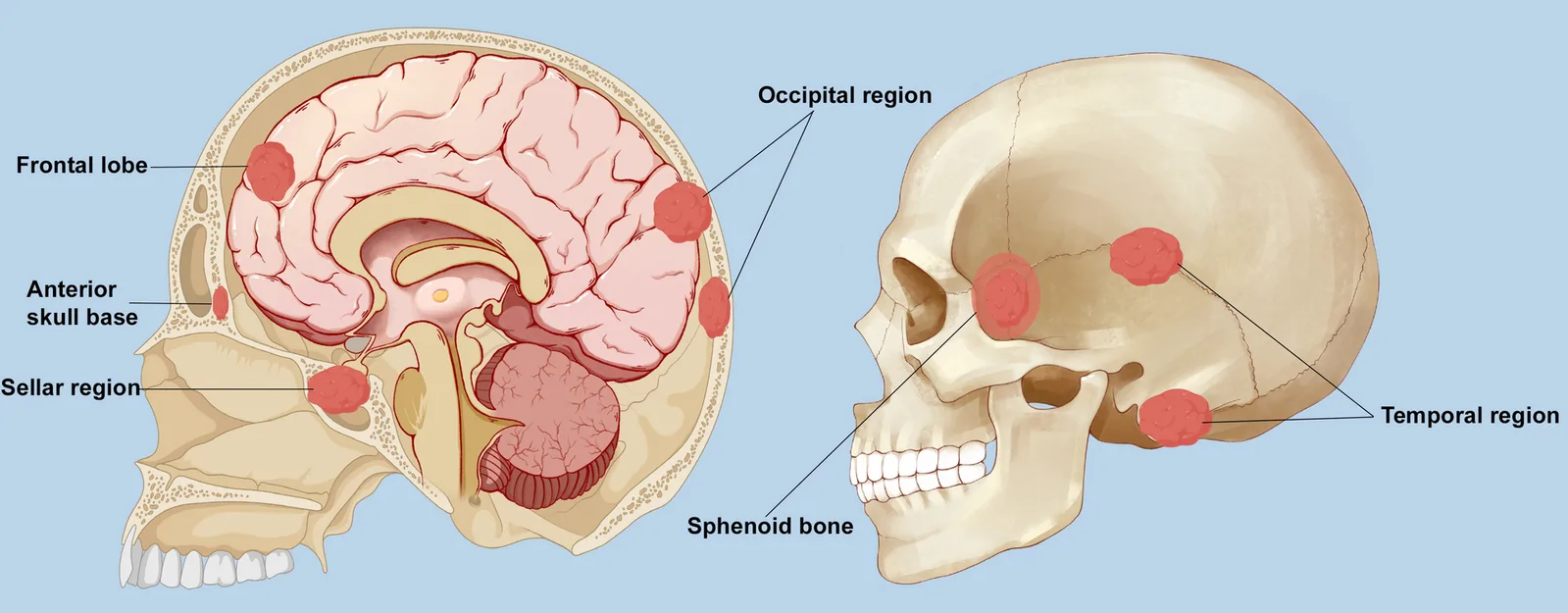
Distribution of TIO tumor in the NC and IC structures by Figdraw

13 TIO patients with lesions in the NC and IC structures had been treated in our center. The locations of their lesions were shown in Table 2; Fig. 4. The anterior skull base (23.1%) and the temporal region (38.4%) were more common locations. The lesions of temporal region often involved the ear canal, therefore some patients began with hearing loss. TIO lesions could also appear in the sellar region (7.7%), frontal lobe (7.7%) occipital lobe (15.4%) and sphenoid bone (7.7%).

**Table 2:** Distribution of TIO tumor in the NC and IC structures

Tumor locations	No. (%)
Sellar region	1/13 (7.7%)
Frontal lobe	1/13 (7.7%)
Occipital region	2/13 (15.4%)
Sphenoid bone	1/13 (7.7%)
Anterior skull base	3/13 (23.1%)
Temporal region	5/13 (38.4%)

Categorical variables were expressed as counts/ totality (proportion)

### The tissue source of tumors and the outcomes of pathology

TIO tumors located in the NC and IC structures (83.3%) and in the sinuses (97.6%) were predominantly soft tissue tumors. Majority of tumors in two groups (lesions in the NC and IC structures: 92.3%, lesions in the sinuses: 92.9%) were typical PMTs. Although it was not statistically significant, Ki67 in lesions in the NC and IC structures [7.50 (1.25, 13.75)] was higher than that in lesions in the sinuses [3.00 (1.75, 5.00)] in the existing cases. There were no significant differences in the remaining immunohistochemical results between the two groups (Supplement Table 1) [7].

### Outcomes of TIO patients with lesions in the NC and IC structures and lesions in the sinuses

**Fig. 5:**
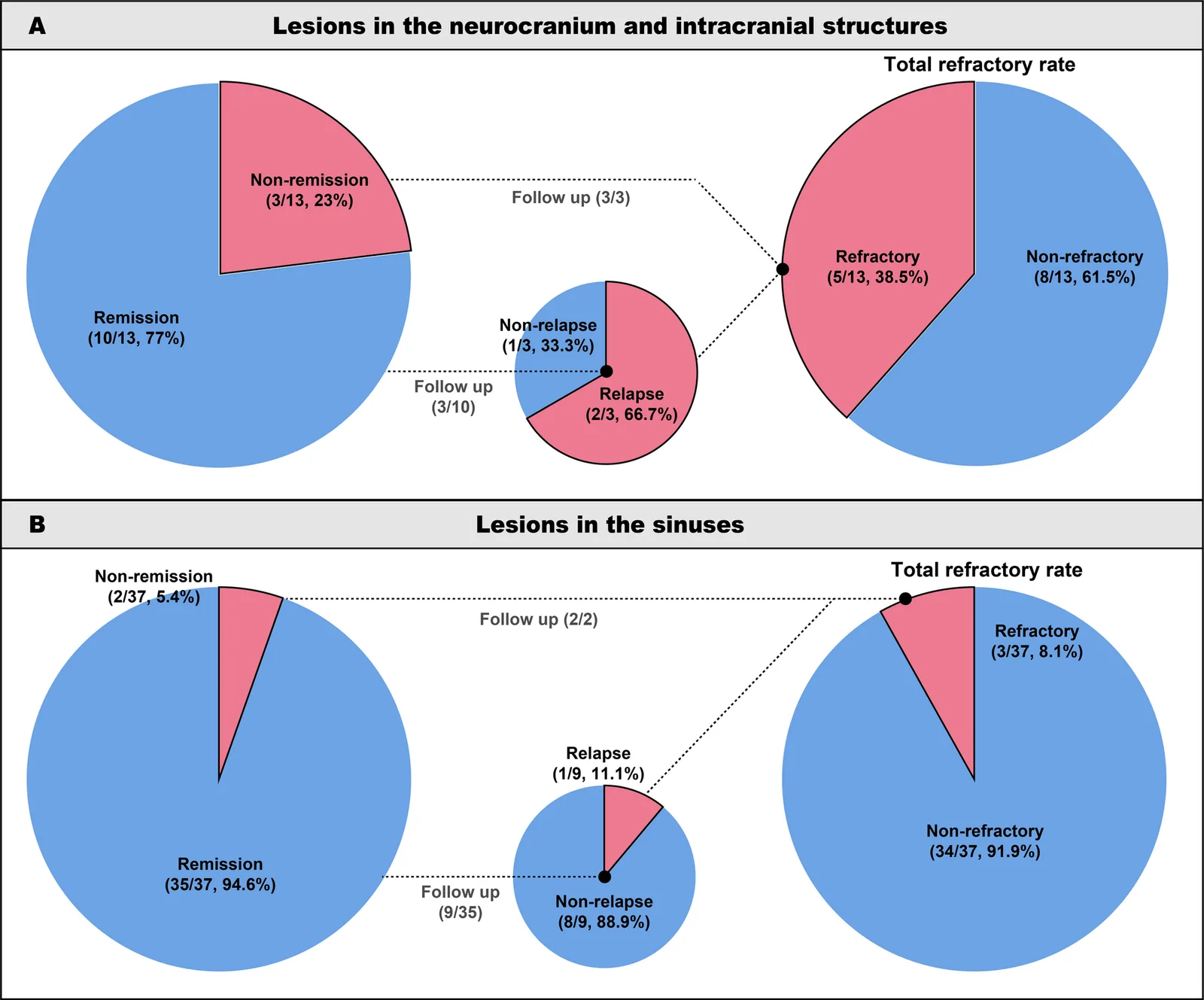
Outcomes of TIO patients. (**A**) Outcomes of TIO patients with lesions in the NC and IC structures. (**B**) Outcomes of TIO patients with lesions in the sinuses

TIO patients with lesions in the NC and IC structures (23.1%) had a higher non-remission rate compared to TIO patients with lesions in the sinuses (5.4%). Among patients who had recovered and had follow-up data, TIO patients with lesions in the NC and IC structures (66.7%) had a higher relapse rate compared to TIO patients with lesions in the sinuses (11.1%). Therefore, according to our definition of refractory, the refractory rate of TIO patients with lesions in the NC and IC structures (38.5%) was also higher than TIO patients with lesions in the sinuses (8.1%) (Fig. 5).

### Postoperative condition of TIO patients with lesions in the NC and IC structures

**Fig. 6:**
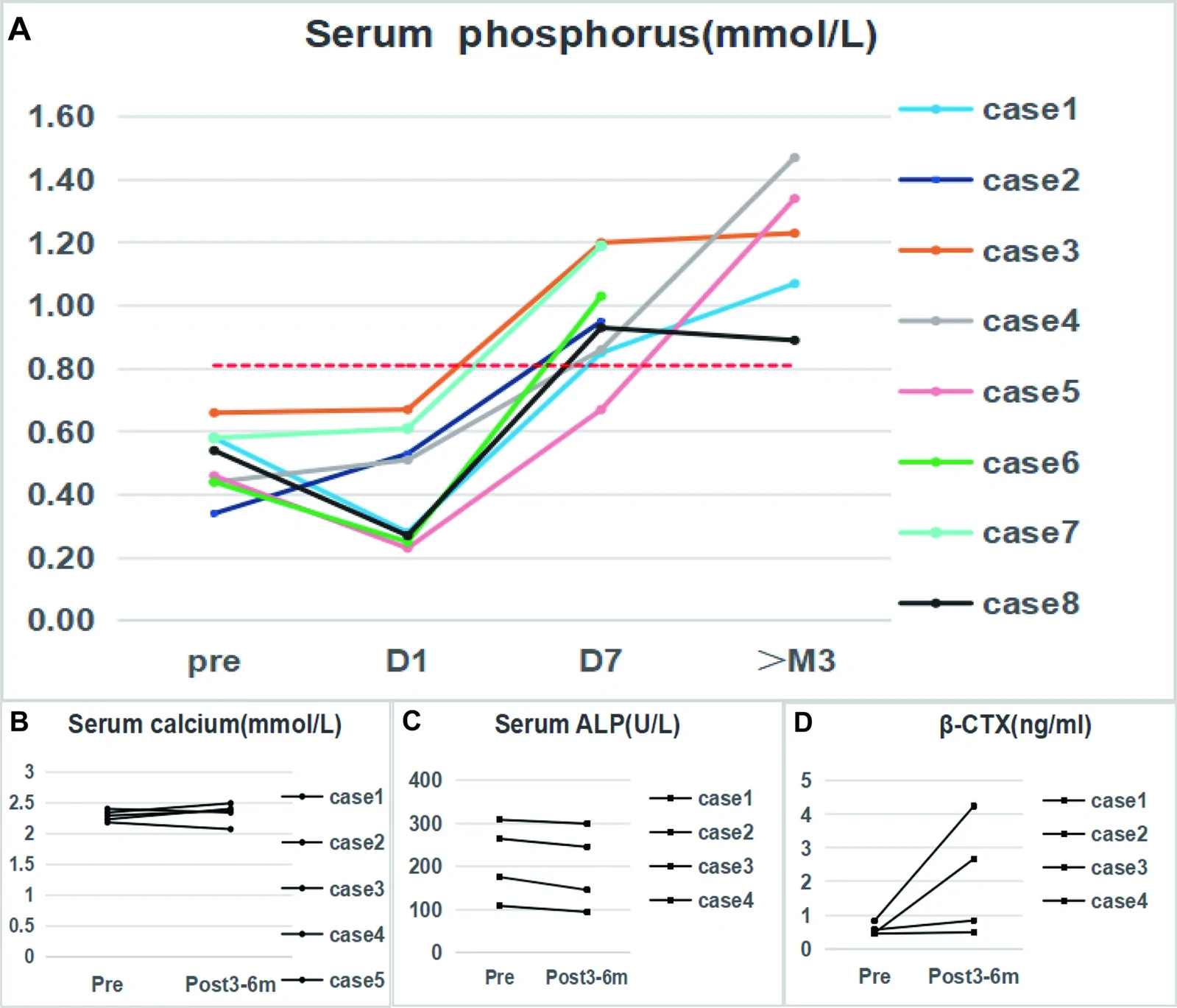
Postoperative changes of biochemical parameters. (**A**) Postoperative changes of serum phosphorus at D1, D7, > M3 (**B**) Postoperative changes of serum calcium at 3–6 months (**C**) Postoperative changes of serum ALP at 3–6 months (**D**) Postoperative changes of β-CTX at 3–6 months

In TIO patients with lesions in the NC and IC structures who have recovered postoperatively and have follow-up data, the changes in biochemical parameters before and after surgery were shown in Fig. 6. 87.5% of the patients’ serum phosphorus returned to normal levels by the 7th day postoperatively, while there was still one patient whose serum phosphorus had not returned to normal by the 7th day postoperatively. Serum calcium levels were within normal limits before and after surgery and showed no significant changes. Serum ALP levels decreased somewhat between the 3rd and 6th months postoperatively compared to preoperative levels, but the decrease was not significant and did not return to normal levels. Only one patient had normal ALP levels both before and after surgery. The level of β-CTX significantly increased between the 3rd and 6th months postoperatively compared to preoperative levels.

## Discussion

TIO is a rare paraneoplastic syndrome characterized by excessive production of FGF23 by tumors, leading to increased renal phosphate excretion and significant hypophosphatemia. Patients typically present with bone pain, muscle weakness, and fragility fractures [1, 2, 4]. TIO lesions in the NC and IC structures represent a relatively rare subtype of TIO tumors. This study summarized all cases of TIO lesions in the NC and IC structures in the largest Chinese TIO cohort to date. To better understand the characteristics of these lesions, we compared them with TIO lesions in the sinuses, due to their anatomical proximity and the fact that some sinus lesions invaded the skull base. Our findings revealed that TIO lesions in the NC and IC structures were not only anatomically distinct but also exhibited unique clinical characteristics compared to other TIO lesions in the head and neck region. These insights underscore the necessity for developing more tailored clinical treatment strategies based on the specific features of these lesions.

In addition to typical TIO symptoms like fatigue and bone pain, patients with lesions in the NC and IC structures developed unique neurological symptoms such as hearing loss and restricted eye movement due to the lesions’ locations. The rarity and subtle onset of TIO led to frequent misdiagnosis, often delaying accurate diagnosis by years, resulting in severe bone symptoms [8]. These neurological symptoms served as early warnings, prompting patients to seek further evaluation in neurosurgery or otolaryngology. The results of the postoperative pathology allowed for retrospective diagnoses of TIO. Consequently, the disease course for patients with lesions in the NC and IC structures was typically shorter compared to those with lesions in other locations [9]. Complete tumors resection, while currently the only definitive cure for TIO, carried the risk of brain dysfunction due to special locations of the lesions. In our study, 4 out of 13 patients with lesions in the NC and IC structures had preoperative compression symptoms. Preoperative symptoms in these 4 patients persisted postoperatively. Among the 9 without preoperative symptoms, 2 developed complications postoperatively due to the tumors’ locations, highlighting the need for advanced surgical skills.

In this study, we summarized the locations of TIO lesions in the NC and IC structures. Previous research from our otolaryngology department reported 43 cases of TIO with sinus lesions, including 12 involving the skull base [10]. Combining previous clinical experience with the findings of this study, the skull base could been identified as a common site for TIO lesions in the NC and IC structures. The temporal region also emerged as a frequent site, with tumors potentially located in the temporal bone, mastoid, or ear canal due to its complex anatomy. While lesions in the sella, frontal lobe, and occipital region were relatively rare, this study underscored the possibility of their occurrence. Due to limited clinical experience with TIO in these locations, a patient with a frontal lobe TIO lesion was initially misdiagnosed with meningioma, while another patient with limited eye movement and reduced anterior pituitary function had a 5 cm sellar lesion initially also misdiagnosed as meningioma. Through multidisciplinary discussions that incorporated clinical symptoms and functional examination results, these two lesions were eventually identified as possible TIO lesions, and finally confirmed by surgery. These cases have expanded our diagnostic and therapeutic knowledge of TIO lesions above, offering significant reference value for clinical practice.

Patients with TIO lesions in the NC and IC structures exhibited higher Ki67 levels compared to those with sinus lesions. Additionally, the non-remission, relapse, and refractory rates of TIO lesions in the NC and IC structures were all higher than TIO lesions in the sinuses, with a refractory rate of 38.5%, which deviated from previous findings. In previous studies from other centers, the refractory rate of TIO lesions in the head and neck was about 14.3% [11–16]. Our center’s data showed a lower refractory rate of 7.5% for head and neck lesions, 23.1% for hip and pelvis lesions, and 77.8% for spinal lesions [4]. Previous studies often grouped TIO lesions in the NC and IC structures with those in the sinuses and jaws. However, our findings suggested that TIO lesions in the NC and IC structures had distinct anatomical and clinical characteristics. Therefore, it was crucial to analyze these lesions separately to avoid underestimating their complexity. For a patient with a lesion in the occipital region, preoperative anatomical imaging indicated potential local bone invasion. Considering the high refractory rate of such lesions, the surgical plan incorporated the possibility of replacing the occipital bone with a titanium alloy, which was thoroughly discussed with the patient. During surgery, extensive local invasion was confirmed, necessitating the bone replacement. The careful preoperative planning and execution resulted in a successful outcome, highlighting the need for a comprehensive understanding of TIO lesions in the NC and IC structures to refine treatment strategies.

In patients with postoperative recovery, most had serum phosphorus levels normalize by the seventh day, though some levels remained low. Given the complexity of lesions in the NC and IC structures, a longer period may be required for resuming normal eating. Therefore, retesting serum phosphorus levels when the patient was in better overall condition was recommended. TIO patients with lesions in the NC and IC structures showed decreased ALP levels and increased β-CTX levels 3 to 6 months postoperatively, indicating active bone turnover and repair. These findings were consistent with previous studies on TIO patients [10, 17].

Since the major pathogenic mechanism of TIO in different locations were similar, we found no significant difference in general clinical characteristics and biochemical parameters between TIO patients with lesions in the NC and IC structures and those with lesions in the sinuses as theorized. In this study, there was also no statistical difference in the positive rate of ^99m^Tc-OCT and ^68^Ga-DOTA-TATE PET/CT between lesions in the NC and IC structures and in the sinuses.

This article still had certain limitations. First, although this cohort was currently the largest TIO lesions in the NC and IC structures cohort, it had improved our understanding of such lesions to some extents, the total number of patients was still small. Second, there was a lack of postoperative follow-up data for FGF23, ALP, β-CTX, etc., making the understanding of the disease’s postoperative outcome incomplete. In summary, further expansion of the cohort and more research are still needed.

In conclusion, we reported the clinical characteristics of TIO lesions in the NC and IC structures in the largest Chinese TIO cohort to date. For patients with hypophosphatemic osteomalacia who exhibited cranial compression symptoms, it was important to consider that TIO lesions may be located in the NC and IC structures. In addition to the skull base and temporal region, TIO lesions could also occur in the sellar region, frontal lobe, and occipital region. In patients with cranial tumors who had hypophosphatemia, the tumor should be considered as a possible TIO lesion. The refractory rate of TIO lesions in the NC and IC structures was higher than TIO lesions in other locations except spine. Therefore, in the treatment process of such lesions, both the special anatomical location and the special clinical characteristics should be emphasized to provide patients with better treatment strategies.

## Supplementary Information

Below is the link to the electronic supplementary material.

Supplementary Material 1

## Data Availability

The datasets used and/or analysed during the current study are available from the corresponding author on reasonable request.
