# Supplementary material for: Clinical characteristics of tumor-induced osteomalacia lesions in the neurocranium and intracranial structures
Source: Orphanet J Rare Dis. 2026 Jul 7;21:341. doi: 10.1186/s13023-026-04477-1 (PMC13637145; doi:10.1186/s13023-026-04477-1)
Supplement: Supplementary file 1 [file 13023_2026_4477_MOESM1_ESM.docx]

**Supplement Table 1. The tissue source of tumor and the outcomes of pathology and immunohistochemistry of TIO patients with lesions in the NC and IC structures and lesions in the sinuses**

|  | **lesions in the NC**  **and**  **IC structures** | **lesions in the sinuses** | ***P*** |
| --- | --- | --- | --- |
| **The tissue source of tumor** | | | |
| Soft tissue, n (%) | 10/12 (83.3%) | 40/41 (97.6%) |  |
| Bone involved, n (%) | 2/12 (16.7%) | 1/41 (2.4%) |  |
| **Pathology** | | | |
| PMT or PMTMCT | 12/13 (92.3%) | 39/42 (92.9%) |  |
| Non**-**PMT | 1/13 (7.7%) | 3/42 (7.1%) |  |
| **Immunohistochemistry** | | | |
| Ki67 | 7.50 (1.25, 13.75) | 3.00 (1.75, 5.00) | 0.353 |
| CD56+ | 4/4 (100%) | 8/9 (88.9%) |  |
| CD34+ | 3/5 (60%) | 8/9 (88.9%) |  |
| BCL**-**2+ | 4/4 (100%) | 8/9 (88.9%) |  |
| CD99+ | 4/4 (100%) | 6/9 (66.7%) |  |
| Vimentin+ | 3/3 (100%) | 9/9 (100%) |  |
| NSE+ | 3/4 (75%) | 8/9 (88.9%) |  |
| S-100+ | 1/3 (33.3%) | 0/0 (0%) |  |
| Synaptophysin+ | 2/2 (100%) |  |  |
| SMA+ |  | 0/1 (100%) |  |
| Desmin+ | 0/2 (100%) | 0/2 (100%) |  |
| AE1/AE3+ | 0/5 (0%) | 1/9 (11.1%) |  |
| D2**-**40+ | 2/3 (66.7%) | 4/8 (50%) |  |

Non-normally distributed continuous variables were shown as median (interquartile range, IQR). Categorical variables were expressed as counts/totality (proportion).

Abbreviations: PMT, phosphaturic mesenchymal tumor.
